# Use of Growth Hormone in the IVF Treatment of Women With Poor Ovarian Reserve

**DOI:** 10.3389/fendo.2019.00500

**Published:** 2019-07-24

**Authors:** Roger J. Hart

**Affiliations:** ^1^Division of Obstetrics and Gynaecology, University of Western Australia, Perth, WA, Australia; ^2^Fertility Specialists of Western Australia, Bethesda Hospital, Claremont, WA, Australia

**Keywords:** growth hormone, IVF, ovarian reserve, poor responder, embryo quality

## Abstract

Growth hormone (GH) has been used as an adjunct in the field of female infertility treatment for more than 25 years, although, apart from treating women with GH deficiency its role has not yet been clarified. Contributing to this lack of clarity is that several underpowered studies have been performed on women undergoing IVF treatment, with a previous “poor response” to ovarian stimulation, which have suggested a favorable outcome. Meta-analysis of randomized controlled trials has demonstrated a benefit for the use of the adjunct growth hormone, in comparison to placebo; with reductions in the duration of ovarian stimulation required prior to oocyte retrieval, with a greater number of oocytes collected, and improvements in many of the early clinical parameters with the use of GH. However, no benefit of an increased chance of a live birth with the use of growth hormone for the “poor responding” patient has been determined. Consequently the role of GH to treat a woman with a poor response to ovarian stimulation cannot be supported on the basis of the available evidence. However, the place for GH in the treatment of women undergoing IVF may yet still be determined, as it is also used, without firm evidence of benefit; for women with poor embryonic development, poor endometrial development and for women who do not conceive despite multiple embryo transfers (recurrent implantation failure).

## Introduction

Growth hormone (GH) is a peptide hormone secreted from the pituitary gland in response to growth hormone releasing hormone, and its secretion is inhibited by growth hormone inhibiting hormone (somatostatin) released form the hypothalamus into the hypophyseal portal system surrounding the pituitary gland. GH is released from the somatotroph cells of the anterior pituitary, with a frequency and amplitude that changes during the day. Its peak secretion occurs after sleep commences, and is age and sex dependent, with maximal secretion occurring around puberty, and its pulsatile release is further modulated by sleep patterns, diet, exercise and stress. As GH is not fat soluble; it exerts its effects via secondary messengers. The main end-point of GH activity is the liver, where GH leads to the synthesis of insulin- like growth factor 1 (IGF-1), which is consequently the major method of action of GH.

In the ovary IGF-1 receptors are present within oocytes, granulosa, and theca cells (1). With respect to the involvement of GH in ovarian function, Zhou et al. (2) demonstrated that IGF-1 acts via its receptor (IGF-1R) in the granulosa cells of the ovarian follicles to stimulate AKT and gene expression by follicular stimulating hormone (FSH). IGF-1 facilitates, or potentiates, the action of FSH in folliculogenesis, and assists with granulosa cell differentiation, with the largest follicles containing the highest concentrations of IGF-1 (2). Studies using murine models suggest IGF-1 is integral to follicular recruitment (3), assists in acquisition of FSH receptors and oocyte maturation (4), and the inhibition of follicular apoptosis (5, 6). Studies performed on human granulosa cells demonstrate that GH co-treatment induces the receptor density of key regulators of folliculogenesis, when compared to the granulosa cells of non-GH-treated patients of the same age and ovarian reserve (7). These key regulators consist of the receptors for: GH, FSH, luteinising hormone, and the receptor of an important regulator of oocyte development; bone-morphogenic protein 1B.

Evidence derived from human clinical observational data provides a rationale for the use GH in *in-vitro* fertilization (IVF) treatment. As the follicular fluid IGF-1 concentrations of women, at oocyte retrieval, are proportional to the number of developing follicles, and are inversely related to the ovarian stimulation required, both in amount and duration (8). Furthermore, follicular fluid concentrations of GH have been correlated with the chance of a clinical pregnancy (9), and the follicular concentration of GH has been reported to be greatest in the follicle that leads to successful oocyte fertilization, embryo development and implantation (10).

As GH was believed to be integral to folliculogenesis, it has been used as an adjunct in ovulation induction, and IVF, for over 25 years (11). It is an essential requirement in the treatment of infertility for women with GH deficiency, as many such women will have a disorder of ovulation (12, 13). Indeed, in line with the study by Zhou et al. (2), Homburg et al. (14) performed a randomized controlled trial of women undergoing ovulation induction, which demonstrated that the requirement for gonadotrophins was reduced for women who were co-administered GH during ovarian stimulation.

As described, GH has been used for many years in the treatment of female infertility to assist with ovulation induction (14), however, it is the use of GH as an adjunct in IVF treatment where most debate has occurred (15). GH has been employed for all women embarking on IVF treatment (16, 17), for women with polycystic ovary syndrome (18, 19), for women responding sub-optimally to ovarian stimulation in an IVF cycle (20), for “older” women (7), and for women with perceived poor oocyte or embryo quality (21). Although, interestingly it is perhaps not as a therapy to improve ovarian response, or oocyte quality, where any benefit of GH may lie, as a recent Chinese study suggests it may offer a favorable benefit for women undergoing IVF treatment who have a thin endometrium resistant to any therapy (22).

Although lacking FDA approval for its use in an IVF cycle, other than in the setting of GH deficiency, GH is most commonly used as an adjunct to ovarian stimulation for women who had a poor response to ovarian stimulation in a preceding IVF cycle. Despite the 25 years of use of GH to assist in the treatment of female infertility, its role in IVF treatment is still debated today (15). This is in part due to the problems inherent with the reporting of underpowered studies of patients with a poor prognosis for pregnancy. The reason these women, with a poor prognosis for pregnancy, have a low chance of conceiving is that they may well have already undergone several unsuccessful IVF treatment cycles, and are perceived to have either a poor response to stimulation, or suffer with poor oocyte quality. Furthermore, difficulty arises in determining whether GH has a role in the treatment of female infertility as; the drug is expensive, it is unclear what is the appropriate dose to use, when the GH treatment should be commenced, or even in which sub-group of patients it should be used (15). However, the focus of this review is the use of GH for women with a poor ovarian reserve. Poor ovarian reserve is now generally classified by using a standardized definition, the Bologna criteria is the definition most widely embraced (23); requiring two of the following features:

- advanced maternal age (≥40 years) or other risk factors for poor ovarian response
- a previous poor ovarian response (≤ 3 oocytes with a conventional stimulation protocol)
- an abnormal ovarian response test (antral follicle count <5–7 or anti-Müllerian hormone <0.5–1.1 ng/ml [<3.6–7.9 nmol/l]).

Although this definition has its detractors (24), it is useful to employ standard definitions when studying populations, as this enables comparisons with other studies performed in similar sub-sets of women undergoing fertility treatment, although there may be other subtle differences that may arise related to other differences, such as ethnicity. However, despite the introduction of this standardized definition, the majority of the studies of the use of GH were performed prior to the introduction of this definition. Consequently, each study has differing inclusion criteria for the subjects studied.

## The use of Growth Hormone for Poor Responders Undergoing IVF Treatment

The first randomized controlled trials of the use of GH as an adjunct for all women undergoing IVF treatment, were performed two decades ago by Tapanainen (16) and Younis (17). These studies did not detect any differences in any clinical parameter studied with the addition of GH (16, 17). Subsequently the majority of the studies of the use of GH in IVF treatment have been restricted to women who respond poorly to ovarian stimulation (15, 20, 25–28). Despite an apparent benefit noted when using GH, for some of the clinical parameters studied, the studies have been characterized by substantial differences in the inclusion criteria, and differences in the dose and timing of the GH administration, leading to a lack of clarity around any potential benefit.

The largest randomized study performed to date was an open label study performed by Dakhly et al. (28). This study included 240 women who met the Bologna criteria for poor ovarian response, who undertook an IVF cycle using a “long-down regulation” protocol. This approach initiates pituitary desensitization with the use of a gonadotrophin releasing hormone analog commenced prior to ovarian stimulation. The patients in the active arm commenced 7.5 IU GH at the start of pituitary down-regulation, which due to the purported method action of GH has a sound rationale (29). The primary outcome was the live birth rate, using all available fresh and frozen embryos generated. Consistent with previous studies, this group demonstrated a benefit with the use of GH, with respect to; a shorter duration of stimulation, less FSH requirement, a higher serum oestradiol concentration at oocyte retrieval, more oocytes collected, the development of more fertilized oocytes and more embryos generated. However, ultimately there was no difference in the cumulative live birth between the two groups 18.3 vs. 14.7%, for use of GH vs. control respectively (28).

The most recent published randomized trial performed to date is the Australian multi-center “LIGHT” study (30). Non-obese women of 40 years of age, or younger, were eligible for inclusion if they had responded poorly in at least one previous IVF cycle (≤5 oocytes collected), on a high dose of ovarian stimulation. They were excluded if they had a recorded serum FSH concentration >15 IU/l. They were randomized to receive either; 12iu GH from the day of ovarian stimulation, or placebo, which was administered daily in a double-blind protocol (30). The majority of embryo transfers were of a single blastocyst, and the data was analyzed by intention to treat analysis. Of those women that achieved an oocyte retrieval, there was no difference in the chance of a live birth between those women that were administered GH, and those that received the placebo (14.5 vs. 13.7%). However, more women achieved an oocyte retrieval in the GH group, 95.4%, in comparison to 78.5% of women in the placebo group, and they had, on average, one more oocyte collected (5 vs. 4 oocytes). Although, there were no differences in the chances of the women in the GH group reaching an embryo transfer, and there were no differences detected in embryonic development between the two groups (30).

**Figure 1:**
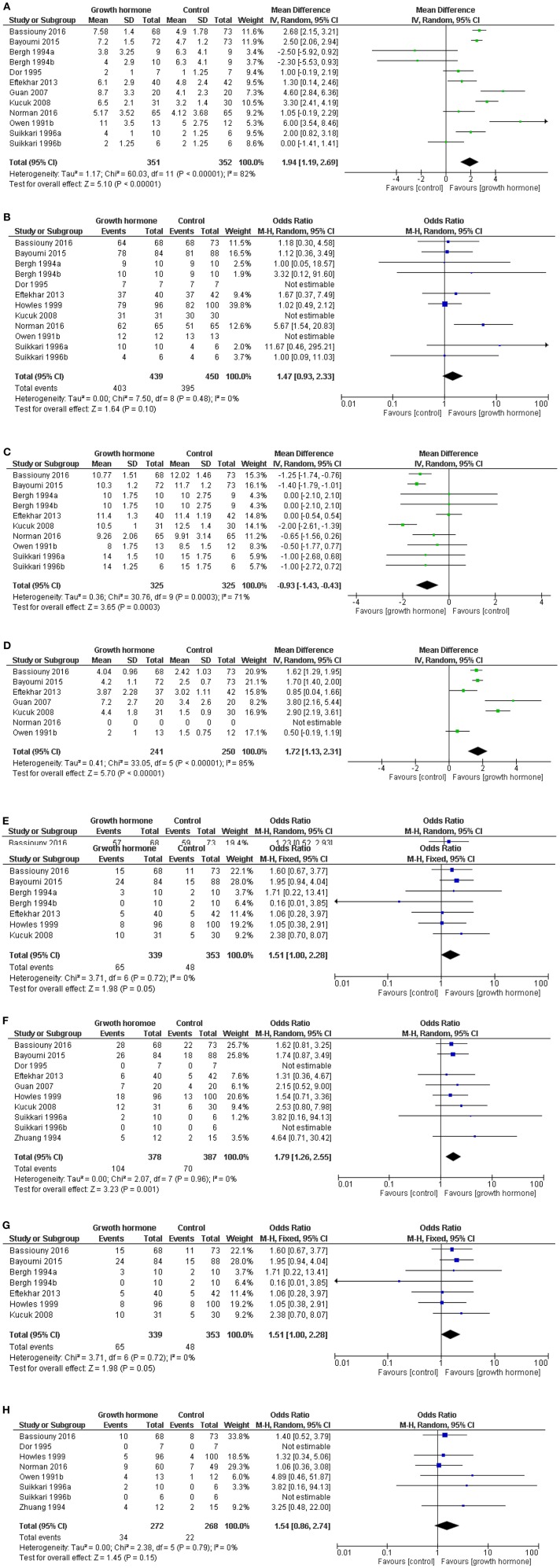
Meta-analysis of the use of GH in poor responders. Forest plots of outcomes from the use of growth hormone in “poor responders” undergoing ovarian stimulation. Where possible data presented per cycle started (median and range converted to mean and std. dev.) Software RevMan Version 5.3. Copenhagen: The Nordic Cochrane Centre, The Cochrane Collaboration, 2014. Reproduced with permission from Hart et al. (15). **(A)** Forest plot of comparison: oocytes collected per cycle started. Not all patients reached oocyte retrieval. **(B)** Patients reached oocyte retrieval and had at least one oocyte retrieved. **(C)** The duration of stimulation. **(D)** Number of fertilized oocytes for women per cycle started (some data is presented by patients who had oocytes retrieved). **(E)** Patients with an embryo available for transfer per cycle started. **(F)** Positive pregnancy test per cycle started. **(G)** Clinical pregnancy per cycle started. **(H)** Live birth per cycle started (15, 31–41).

Using data from the LIGHT study, but without the inclusion of the Dakhly study, the most up to data meta-analysis was published in 2017 (15) (Figures 1A–H). The data within the meta-analysis was derived from studies that used heterogeneous definitions of a “poor responder,” and the doses, timing, and duration of administration of GH varied substantially (31–41). Furthermore, some studies did not report their data per cycle started, as many patients will not have achieved an oocyte retrieval or an embryo transfer, leading to a potential bias in the results (15). This meta-analysis did not demonstrate a benefit of the chance of proceeding to an oocyte retrieval, however there was a benefit of the use of GH with respect to a shorter time taken to oocyte retrieval and an increased number of oocytes were collected. More oocytes achieved fertilization with the use of GH, but there was no increase in the chance in having an embryo to available to transfer. More patients had a positive pregnancy test after GH administration, and achieved a clinical pregnancy, but there was no overall improvement in the live birth rate reported in this meta-analysis (15).

## Discussion

For women who respond poorly to ovarian stimulation in an IVF cycle, it appears that there is no overall benefit of the use of GH, as GH does not lead to an improvement in the chance of a live birth. Live birth is the ultimate end-point of relevance, and should be considered the primary outcome of all infertility interventions. Certainly it would appear from the evidence cited, that the use of GH leads to a reduced time to oocyte retrieval and more oocytes collected, although ultimately no increase in the chance of a live birth. Understandably, in clinical practice, clinicians and patients alike, are encouraged by the opportunity to have more oocytes collected with the use of GH, as they may perceive this as giving them the potential of a more successful outcome. This leads to the inherent uncertainty of the role of GH in IVF treatment, as patients are often in a vulnerable position being desperate to conceive, and the opportunity of having more oocytes collected to them is viewed positively, and consequently they may apply pressure on their IVF doctor to prescribe GH. Although the use of GH has a good safety profile, it is very expensive. Hence, the treatment is for many cost prohibitive and many patients will not be able to afford the treatment. For patients willing to pay for the intervention, doctors should use GH under caution, as IVF is not a defined indication for its use, and the evidence cited does not support it use for women with a poor response to ovarian stimulation. It is important to state that as GH use is “off-label” couples are warned of potential unknown consequences for the offspring. Indeed the authors of the LIGHT study stated “While congenital abnormalities were not different between groups, the number in the growth hormone treatment group warrants ongoing surveillance of treatment with this hormone.”

A concern that is as yet unanswered, is the discrepancy between the favorable influences on pregnancy rates that is not reflected in live birth rates. It is unclear as to whether the studies performed are underpowered for live birth outcome data, or whether it is that GH may just recruit an oocyte that is poor in quality and subsequently results in an early pregnancy loss. Only further studies will help to clarify this apparent dichotomy.

However, GH may not have found its appropriate indication, as it may be that GH assists a sub-group of poor responder patients; women with poor oocyte and/or poor embryonic development (21). However, no randomized studies have been performed on these sub-groups of patients, perhaps due to the difficulties in defining a “poor oocyte” or “poor embryonic development.” Furthermore, many women will meet the criteria of a “poor responder,” but the populations of women within this criteria can be disparate, for instance, they may differ with respect to; the woman's age (a significant marker of oocyte quality), or they may differ with respect to the number of un-recruited follicles when stimulated. For example, a 40-year-old woman with no antral follicles on ultrasound examination is evidently a different clinical scenario to a woman of 30 years of age with a few un-recruited antral follicles visualized on ultrasound, however they both meet the criteria of a poor responder (23). This has led to clinicians further exploring the definition, and now describing a group of patients as the “sub-optimal responder” (42). Furthermore, it is possible that the indication for GH may not reside with either the poor responding patient or the patient with poor embryonic development; as it may ultimately be used for patients with poor endometrial development, or even in patients with recurrent implantation failure (22, 43–45).

## Conclusion

Growth hormone has been used as an adjunct in fertility treatment for over 25 years, although apart from the treatment of women with GH deficiency its role has still to be clarified. Many underpowered studies have been performed on women with a poor response to ovarian stimulation. While GH almost universally appears to reduce the duration of ovarian stimulation required for oocyte retrieval, and lead to the collection of a greater number of oocytes than women who received a placebo, and many of the early clinical parameters appear favorable; there is no evidence to demonstrate an increased chance of a live birth for a woman who receives GH for this indication. Whether the role of GH resides in the treatment of poor oocyte quality, or to treat the “sub-optimal” responder, or in the treatment of “the thin endometrium” or “recurrent implantation failure” awaits further investigation and clarification.

## Author Contributions

The author confirms being the sole contributor of this work and has approved it for publication.

### Conflict of Interest Statement

RH is the Medical Director of Fertility Specialists of Western Australia and a shareholder in Western IVF, he has received educational sponsorship from MSD, Merck-Serono and Ferring Pharmaceuticals.
